# Comparative Transcriptome Analysis Provides Insight into the Effect of 6-BA on Flower Development and Flowering in *Bougainvillea*

**DOI:** 10.3390/plants14223442

**Published:** 2025-11-10

**Authors:** Xinggu Lin, Hong Chen, Miaomiao Sun, Xuelin Du, Sixian Zeng, Beiyi Guo, Seping Dai, Guofeng Liu

**Affiliations:** 1 Guangzhou Landscape Plant Germplasm Resource Nursery, Guangzhou Institute of Forestry and Landscape Architecture, Guangzhou 510540, China; linxinggu@hotmail.com (X.L.); chenhong2323@163.com (H.C.); smm5662466@163.com (M.S.); d1239508921@163.com (X.D.); cherish2021318@163.com (S.Z.); gzifla_gby@gz.gov.cn (B.G.); kjtgb01@163.com (S.D.); 2 Guangzhou Collaborative Innovation Center on Science-Tech of Ecology and Landscape, Guangzhou Institute of Forestry and Landscape Architecture, Guangzhou 510540, China

**Keywords:** *Bougainvillea glabra*, 6-BA (6-Benzylaminopurine), transcriptome analysis, flowering regulation, cytokinin metabolism

## Abstract

*Bougainvillea* spp. is a well-known ornamental plant that is widely applied in urban landscaping construction. The colorful bracts of *Bougainvillea* in full bloom become important for urban landscape during special festivals. Although flowering regulation measures of *Bougainvillea* attracted much attention, the underlying mechanism of flower bud differentiation and development remains poorly understood. Here, we induced flowering of *Bougainvillea glabra* ‘Sao Paulo’ under 6-BA treatment and conducted RNA sequencing data analysis to characterize the molecular regulatory mechanism of flower development in response to 6-BA. Transcriptome analysis indicated that a series of genes and transcription factors of cytokinin metabolism, flowering and floral development regulation, and photoperiod regulation were upregulated by the 6-BA treatment, including *COL*, *AP2*, *FT*, *SOC1*, *LFY*, *SPL4*, *SPL9*, and *SPL13*. Moreover, the expression of these important genes exhibited relatively high levels of thorns compared to apical buds, suggesting that flower bud differentiation probably starts with the thorns in *Bougainvillea*. This study confirms that 6-BA treatment at certain concentrations can promote flowering of *Bougainvillea* and provides insight into the regulatory mechanism of the growth regulator acting on early flowering of *Bougainvillea.*

## 1. Introduction

*Bougainvillea* spp. (Nyctaginaceae) is a perennial woody plant native to tropical and subtropical areas and is widely cultivated for its vibrant and colorful bracts. Owing to its excellent adaptability and prolonged flowering period, it has become a key species for vertical greening projects in South China. Its application in bridge greening not only expands urban green space but also contributes significantly to carbon sequestration, oxygen release, and the mitigation of air pollution and the heat island effect.

The ornamental appeal of *Bougainvillea* arises from its showy bracts, which surround clusters of inconspicuous perfect flowers [1,2]. The fundamental floral unit comprises a single flower subtended by a bract (Supplementary Figure S1A). Three such basic units constitute a primary cymose inflorescence, known as a bract cluster (Supplementary Figure S1B). Furthermore, three primary cymes combine to form a secondary cymose inflorescence (Supplementary Figure S1C). Multiple whorls of these primary cymes then assemble into cymose panicles of varying sizes (Supplementary Figure S1D). Such an inflorescence structure is ubiquitous in the genus *Bougainvillea*, with the exception of *B. spinosa*, whose floral unit comprises a single flower surrounded by three bracts [2].

Precise control of flowering time is crucial for maximizing bougainvillea’s landscape value during festive seasons. Consequently, various horticultural techniques for flowering regulation, including pruning and shaping, fertilization, water control, light regulation, temperature control, and spraying plant growth regulators (PGRs), have been developed [3,4]. In particular, appropriate concentration of PGRs can effectively promote flower bud differentiation, prolong flowering period and improve ornamental quality. For instance, ethyphon, paclobutrazol (PP_333_), abscisic acid (ABA), and mixed application of ABA and nordihydroguaiaretic acid (NDGA) can promote flower bud formation for early flowering [5,6]. Moreover, gibberellic acid (GA3), naphthalene acetic acid (NAA), GA3 plus NAA, and silver thiosulfate (STS) plus NAA, can increase bract longevity and delay bract abscission, thereby prolonging the anthesis period in *Bougainvillea* [7,8,9]. The synthetic cytokinin 6-benzyladenine (6-BA) has yielded concentration-dependent effects, promoting flowering at high concentrations (200–1000 mg L^−1^) [10], but showing limited efficacy at low doses (30–60 mg L^−1^) [11]. Despite these empirical findings, the molecular mechanisms governing flower bud differentiation and development of *Bougainvillea* remain unclear.

In our preliminary screening of several PGRs for their effects on *Bougainvillea* flowering, including PP_333_, ABA, GA3, 6-BA, chlormequat, ethyphon, and daminozide (B9), 6-BA treatment proved most effective in promoting flower bud differentiation and floral development, resulting in the earliest floral initiation and the highest flower number. Therefore, we selected 6-BA for further investigation into the molecular basis of flowering in *Bougainvillea*.

As the crucial transition from vegetative growth to reproduction, flower bud differentiation represents a fundamental phase in the plant life cycle. It is orchestrated by the integration of internal factors and external environmental cues to ensure successful flowering and reproduction. Flowering in angiosperms is governed by conserved molecular mechanisms, with the genes *FLOWERING LOCUS T* (*FT*), *LEAFY* (*LFY*), *SUPPRESSOR OF OVEREXPRESSION OF CONSTANS 1* (*SOC1*), and *FLOWERING LOCUS C* (*FLC*) playing central roles in *Arabidopsis thaliana* [12,13]. It is noteworthy that the regulatory pathways of exogenous cytokinins in promoting floral transition exhibit species-specific differences. In *Arabidopsis thaliana*, this process primarily involves the upregulation of key flowering genes *TWIN SISTER OF FT* (*TSF)*, *FLOWERING LOCUS D* (*FD)*, and *SOC1* [14]. Whereas in *Crocus sativus*, it depends on the enhanced expression of *LFY* [15].

Although exogenous cytokinins indeed promote flowering in *Bougainvillea*, the underlying molecular mechanisms remain unknown. Based on evidence from cytokinin-induced flowering in other species, we hypothesize that cytokinins promote flower bud differentiation and development in *Bougainvillea* through the specific upregulation of key genes in flowering pathways. To test this hypothesis and elucidate the molecular mechanism of flower bud development under exogenous cytokinin 6-BA treatment, we employed paraffin sectioning, transcriptome analysis, and RT-qPCR across different tissues. This study aims to provide insights into the cytokinin-mediated regulatory mechanism of early flowering in *Bougainvillea* and to identify valuable gene resources for further understanding its flower development.

## 2. Materials and Methods

### 2.1. Plant Materials

Three-year-old cutting plants of *B. glabra* ‘Sao Paulo’ were cultivated in the Germplasm Resource Nursery of Ornamental Plants, Guangzhou Institute of Forestry and Landscape Architecture, Guangzhou, China. At four months before the flowering stage, an experimental group of *B. glabra* ‘Sao Paulo’ was sprayed uniformly onto the leaf surface with 6-BA at a concentration of 1000 mg L^−1^ every seven days, which significantly promoted flowering and increased flower quantity based on our preliminary experiments, while the control group was sprayed with water. Three biological replicates, each with three plants, were applied for each treatment. The second day after the fifth application of 6-BA, apical buds, the sixth thorns away from the apical bud, and the leaves adjacent to the sixth thorns of the 6-BA treatment (6-BA) and control (CK) groups were collected and frozen immediately in liquid nitrogen, then stored at −80 °C.

### 2.2. Paraffin Section

Apical buds and the sixth thorns away from the apical bud of the 6-BA and CK groups were collected and fixed with 50% FAA fixation solution (50% ethanol, 5% acetic acid, and 3.7% formaldehyde) for 48 h. These samples were dehydrated using a gradient of ethanol (30%, 50%, 70%, 83%, 90%, 95%, 100%), setting every gradient for 30 min. Subsequently, the transparency process was carried out sequentially using xylene with different gradients (30%, 50%, 70%, 100%) for 30 min. Moreover, the tissues were infiltrated in xylene for 24 h and then embedded in paraffin. Finally, paraffin-embedded 4 μm thick sections were stained with 0.5% toluidine blue for 30 s and observed using the inverted fluorescence microscope (Nikon DS-Ri2, Tokyo Metropolis, Japan).

### 2.3. RNA Sequencing and Analysis

Total RNA of thorn samples from the 6-BA and CK groups was extracted using the RNeasy Plant Mini Kit (Qiagen, Düsseldorf, Germany). RNA concentration and purity were evaluated on a Qubit^TM^ 4.0 fluorometer (Thermo Fisher Scientific, Waltham, MA, USA). RNA integrity was measured on a Bioanalyzer 2100 system (Agilent Technologies, Santa Clara, CA, USA). A total of 1 μg RNA from each sample was used to construct 150 bp paired-end library for RNA sequencing on the Illumina Nova seq 6000 platform.

The raw sequence data was filtered to remove the adapters and low-quality sequences using Trimomatic version 0.39 software [16]. Then clean data was quality controlled to generate high-quality sequences for subsequent analysis using Perl script of QC_pe.pl (https://github.com/scbgfengchao/, accessed on 10 March 2024) [17]. The high-quality reads were mapped to the index dataset; they were built from the reference genome of *Bougainvillea* [18] using hisat2 version 2.2.1 software (http://ccb.jhu.edu/software/hisat/index.shtml, accessed on 15 March 2024). Subsequently the transcriptome reads were sorted and assembled using stringtie version 2.2.2 software (http://ccb.jhu.edu/software/stringtie/, accessed on 8 May 2024). Then the non-redundant data of each sample set was combined by the merge functions of stringtie version 2.2.2 software. Based on the merged dataset as reference, the FPKM (fragments per kilobase million) value of each gene was calculated to evaluate the relative gene expression.

### 2.4. Identification of Differentially Expressed Genes (DEGs) and Functional Enrichment

Based on the read count data obtained by stringtie software, DEGs between the 6-BA treatment and control groups were identified using DESeq2 version 1.38.3 software [19], setting a *P* correction threshold at 0.05. The statistical GO enrichment and KEGG (Kyoto Encyclopedia of Genes and Genomes) enrichment of DEGs were conducted using the R package ClusterProfiler version 4.6.2 [20]. The significantly enriched terms were determined according to the criteria of the adjusted *p*-value ≤ 0.05.

### 2.5. Reverse Transcription-Quantitative PCR (RT-qPCR)

RT-qPCR of the thorns, apical buds, and leaves from the 6-BA and CK groups was applied to analyze the relative expression levels of candidate genes. Primers for the RT-qPCR analysis were designed using Prime Premier 5.0 software and synthesized by Sangon Biotech (Shanghai, China) (Supplementary Table S1). A total volume of 10 μL of qRT-PCR solution comprised 1 μL of cDNA, 0.4 μM of each forward and reverse primer, 3.2 μL of double-distilled water, and 5 μL of TB Green Premix Ex Taq. Using the *Actin* gene of bougainvillea as a reference [3], the RT-qPCR protocol was described as follows: 94 °C for 30 s and 40 cycles of 94 °C for 5 s and 60 °C for 30 s. The 2^−ΔΔCT^ method was applied to calculate relative expression of candidate genes with three replicates. Significant differences were stated by *t*-test at *p* < 0.05, using the ‘stat_compare_means’ function of R version 4.2.2 software.

## 3. Results

### 3.1. 6-BA Treatment Accelerates Florescence of Bougainvillea

**Figure 1:**
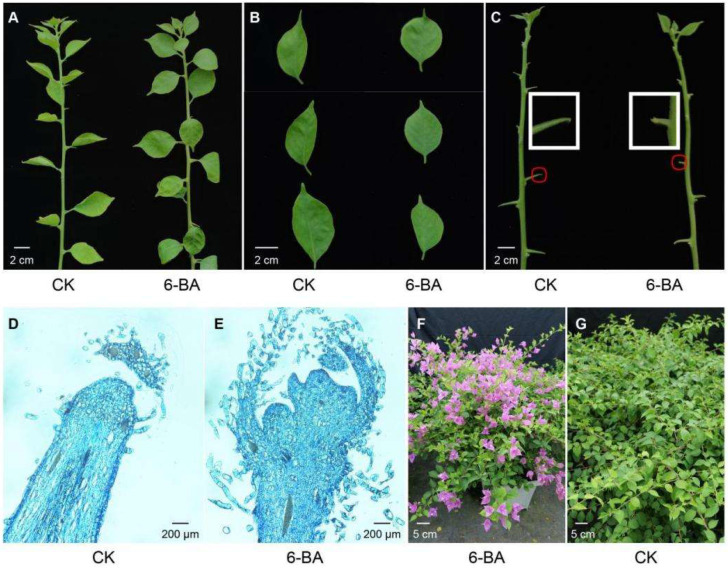
Morphological changes of *B. glabra* ‘Sao Paulo’ between the control (CK) and 6-BA treatment (6-BA) groups. (**A**) The branches of CK (**left**) and 6-BA (**right**) groups. (**B**) The leaves of CK (**left**) and 6-BA (**right**) groups. (**C**) The thorns of CK (**left**) and 6-BA (**right**) groups. The red circles indicate the eighth thorns away from apical bud, and the white boxes correspond to the enlarged view shown. While the control group exhibited wilting at the apical region of thorn, the 6-BA-treated group developed swollen floral buds. (**D**) Paraffin section indicated the apical tissue of thorn for CK group. (**E**) Paraffin section indicated the apical tissue of thorn for 6-BA group. (**F**) Flowering status of 6-BA group after two months of hormone treatment. (**G**) No flowering was observed in the control group after two months.

Compared with the control group, the branches of *Bougainvillea* plants in the 6-BA treatment group exhibited almost no difference, but the leaves showed more roundness, and the tip of the thorns in the axil had obviously developed into flower buds (Figure 1A–C). Paraffin sections of the thorn tissues confirmed that 6-BA treatment promoted floral development in *Bougainvillea*, with significantly promoted growth of floral bud tissues compared to the control group (Figure 1D,E). Under 6-BA treatment, floral buds at the apical region of the thorn exhibited sustained growth and ultimately achieved full bloom (Figure 1F). In contrast, floral bud tissues of the control group displayed lagged development and, subsequently, wilting, abortion, and eventual abscission, resulting in complete failure of flowering (Figure 1G).

### 3.2. Transcriptome Profiling of 6-BA-Treated Thorns and Control Thorns

Raw sequencing reads obtained from each sample ranged from 36.93 million to 53.44 million. After adapters and low-quality sequences removal, high-quality reads with Q30 were greater than 94.7% in rigorous quality control (Supplementary Table S2).

The correlation analysis between the RNA sequencing samples showed that gene expression profiles among samples within the 6-BA and CK groups were much smaller than the differences of the inter-group, indicating the high quality of the biological replicates (Supplementary Figure S2).

### 3.3. Differentially Expressed Genes in Response to 6-BA Treatment

**Figure 2:**
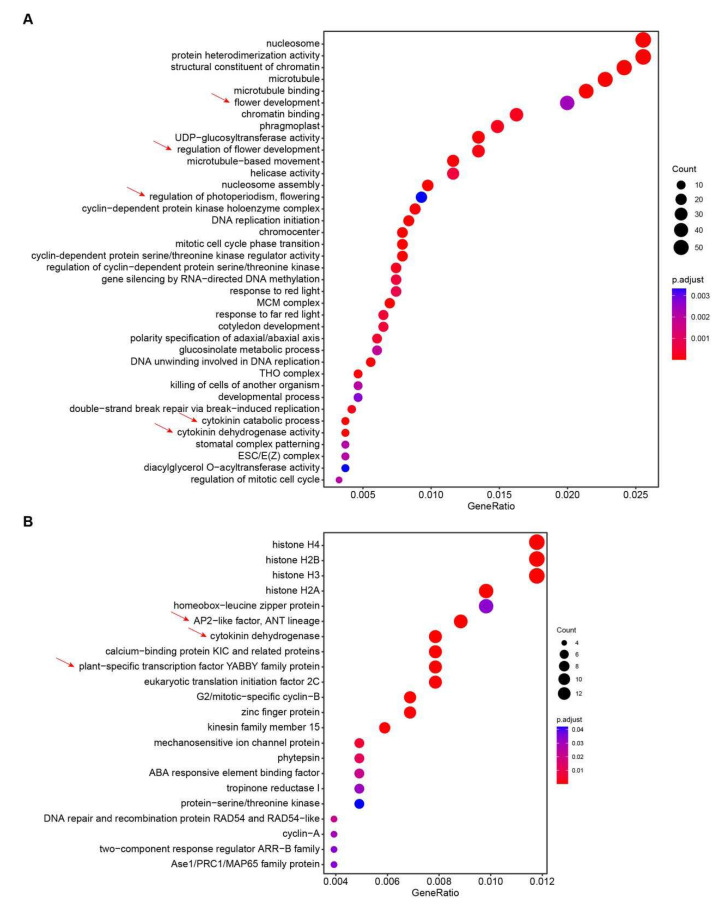
GO enrichment and KEGG enrichment analysis of upregulated differentially expressed genes (DEGs). The vertical axis represents the mainly enriched terms, and the horizontal axis represents the gene ratio. The size of the circle in the figure indicates the total number of enriched DEGs, and the color of the circle indicates their corresponding *p* values. (**A**) GO enrichment dotplot of upregulated DEGs for thorns under 6-BA treatment (6-BA) vs. control group (CK). Red arrows represent the enriched GO terms related to flower development and cytokinin catabolic process. (**B**) KEGG enrichment dotplot of upregulated DEGs between 6-BA and CK groups. Red arrows represent the candidate genes related to flowering enriched in respond to 6-BA treatment.

To explore the effect of 6-BA treatment on flowering of *Bougainvillea*, we performed RNA-Seq analysis using DESeq2 with adjusted *p*-value < 0.05 and log_2_fold-change > |1|, which represented 2-fold-change variations in gene expression level. Then we identified a total of 6456 DEGs from the 6-BA-treated group, including 2730 upregulated genes and 3726 downregulated genes in comparison with the control group (Supplementary Table S3). In order to further investigate the related functions and pathways of upregulated DEGs, GO enrichment analysis suggested that these DEGs were enriched in GO terms of nucleosome, structural constituent of chromatin, cytokinin catabolic process, cytokinin dehydrogenase activity, regulation of flower development, and regulation of photoperiodism related to flowering (Figure 2A, Supplementary Table S4). KEGG enrichment analysis showed that upregulated DEGs were mainly enriched in histone, cytokinin dehydrogenase, AP2-like factor, and YABBY transcription factor (Figure 2B, Supplementary Table S5). In addition, downregulated DEGs were mainly focused on these GO terms of plant-type secondary cell wall biogenesis, the xylan biosynthetic process, and the cellulose biosynthetic process (Supplementary Table S6). These molecular insights are consistent with the phenotypic observations from longitudinal sections and morphological images (Figure 1), which confirmed that 6-BA promotes floral bud development and flowering of *B. glabra* ‘Sao Paulo’. Therefore, we propose that 6-BA exerts its effect by upregulating genes central to cytokinin metabolism, floral development, and photoperiod perception.

### 3.4. Identification of Candidate Genes and Transcription Factors (TFs) During Floral Development

**Figure 3:**
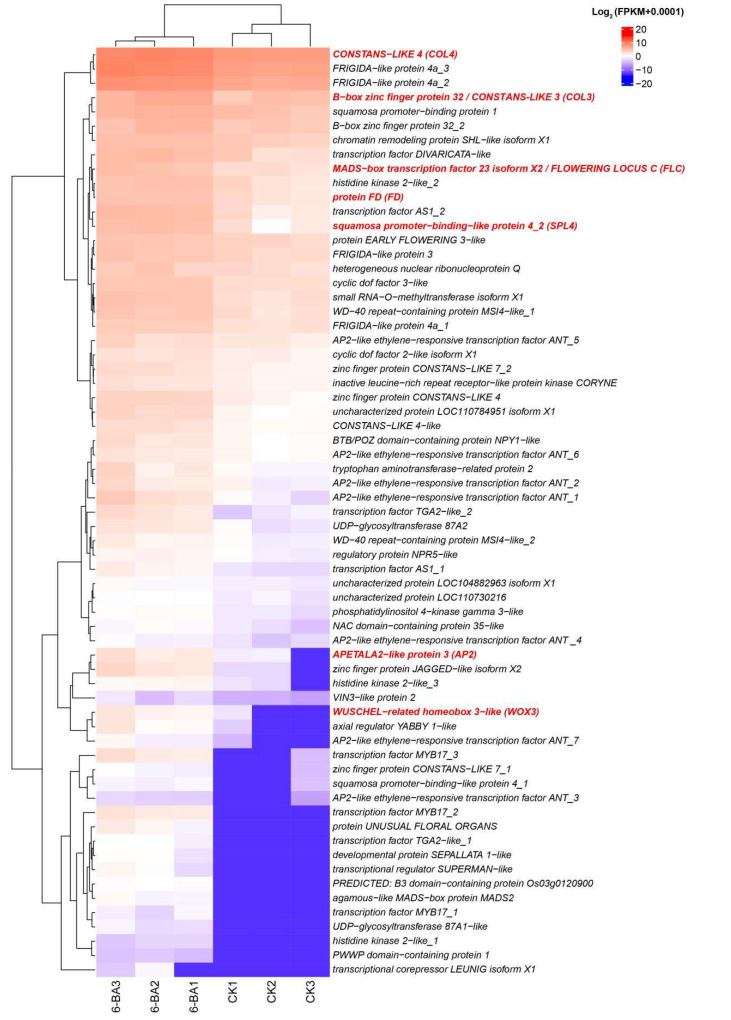
Heatmap cluster of upregulated DEGs enriched in flower development under 6-BA treatment. The genes marked in red were selected for qPCR validation.

As known to all, 6-BA is a common exogenous cytokinin. Consequently, it is reasonable that application of 6-BA induced gene expression changes responding to cytokinin metabolism obviously. For instance, we identified eight cytokinin dehydrogenase (*CKX*) genes that were significantly upregulated under 6-BA treatment. In addition, we found 65 upregulated genes related to flower development (Figure 3, Supplementary Table S7), including *CONSTANS-LIKE* (*COL*), *APETALA2-like* (*AP2*), *FLOWERING LOCUS C* (*FLC*, *MADS-box transcription factor 23 isoform X2*, *Bou_64595*), *WUSCHEL-related homeobox* (*WOX*), *squamosa promoter-binding-like* (*SPL*), *FRIGIDA-like* (*FRI*), and *UNUSUAL FLORAL ORGANS* (*UFO*). Among these genes, nine genes were associated with photoperiodism regulation of flowering (Supplementary Figure S3, Supplementary Table S8), comprising *CONSTANS-LIKE 7*, *WD-40 repeat-containing protein*, and *EARLY FLOWERING*.

**Figure 4:**
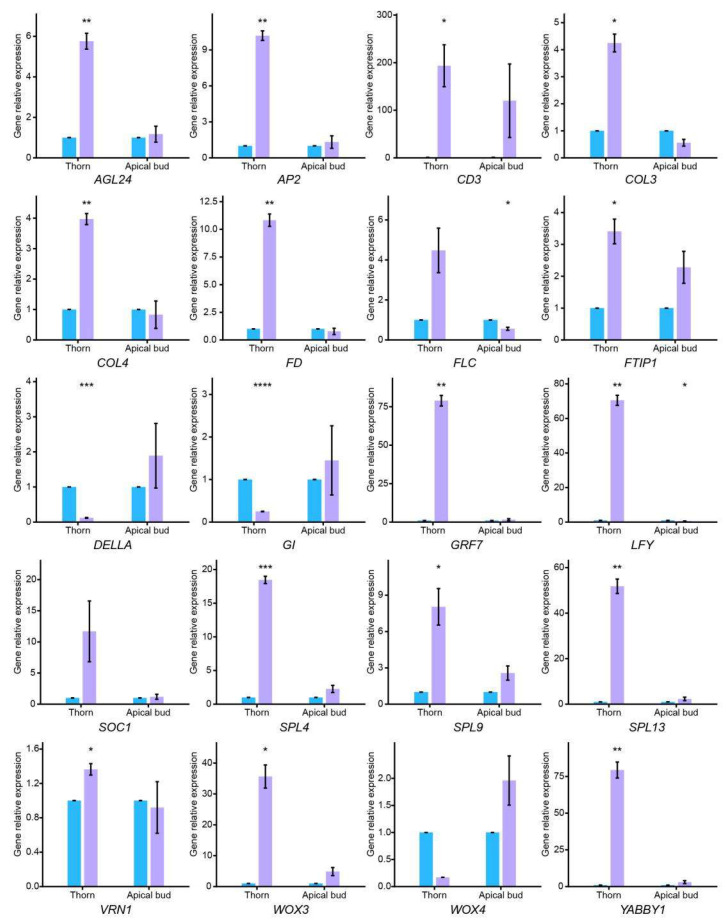
Gene relative expression of flowering-related genes in different tissues using qPCR analysis. Blue and purple bar charts indicate gene relative expression of CK and 6-BA group, respectively. Error bars represent the standard deviation of three biological replicates. Statistical significance between CK and 6-BA group was calculated using the Wilcoxon *t*-test (without *, not significant; * *p* < 0.05; ** *p* < 0.01; *** *p* < 0.001; **** *p* < 0.0001).

To confirm the expression changes of key genes involved in the flowering pathway, real-time qPCR analysis was performed, which verified the gene expression trend of RNA-Seq (Supplementary Figure S4). It is notable that genes *Bou_64595* and *Bou_913* were identified as *FLOWERING LOCUS C* and *FLOWERING LOCUS T*, respectively, with NCBI blast and homologous sequence alignment (Supplementary Figures S5 and S6). Moreover, *Bou_19129* was proved to have a high sequence homology with the *SQUAMOSA* promoter-binding-like gene *SPL13* of *Arabidopsis thaliana* (Supplementary Figure S7). In thorn tissue, floral integrator gene *SOC1*, floral meristem identity gene *LFY*, floral organ identity gene *AP2*, and meristem maintenance gene *WOX3* were significantly upregulated under 6-BA treatment, with the fold change ranging from 10.19 to 70.53 (Figure 4). Moreover, genes related to flower development and flowering, including *AGL*, *SPL*, *FT-interacting protein* (*FTIP*), *VERNALIZATION 1* (*VRN1*), and *growth-regulating factor* (*GRF*) exhibited an upregulated trend. Most of these flowering-related genes showed the same tendency in the apical bud; however, the differences in fold change between the 6-BA treatment and control groups were inconspicuous (Figure 4). These results indicate that exogenous 6-BA can stimulate floral development of *Bougainvillea*, and important genes related to the flowering pathway display more responsiveness in thorn than in apical bud.

## 4. Discussion

*B. glabra* ‘Sao Paulo’ is recognized as a short-day (SD) flowering plant, and its flowering period in Guangzhou is generally from September to December. In this study, we performed the experiment in the unnatural flowering period of May, which could better reflect the effect of treatment on early flowering. We confirmed that 6-BA application could promote flower bud development and flowering of *Bougainvillea.* To investigate the changes in gene expression between the 6-BA group and control group, we performed transcriptomics analysis and identified a series of DEGs associated with flowering pathways and flower development regulation.

Previous research in model plants of *Arabidopsis* has indicated that flowering time is regulated by a variety of environmental and endogenous pathways, mainly including six regulatory pathways: light-dependent pathway, vernalization pathway, temperature pathway, gibberellin pathway, age pathway, and autonomic pathway [12,21]. However, the main pathways to regulate flowering of *Bougainvillea* have not been clearly defined. So far, the flowering regulation of *Bougainvillea* has attracted much attention, but it is limited to some technical exploration of water control, light control, and growth regulators. The molecular mechanism and gene expression changes of flower formation under flowering control techniques remain poorly understood.

**Figure 5:**
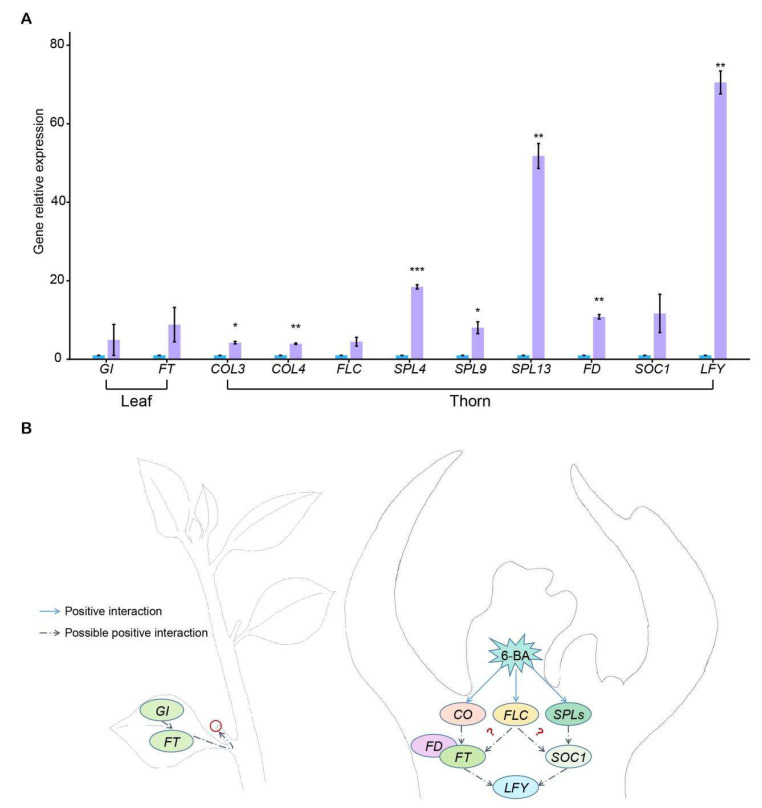
Schematic of the regulatory genes and candidate pathways in flower development of *Bougainvillea* in response to 6-BA. (**A**) Gene relative expression of candidate genes in leaf or thorn tissues by qPCR analysis. Blue and purple bar charts indicate gene relative expression of CK and 6-BA group, respectively. Error bars represent the standard deviation of three biological replicates. Statistical significance between CK and 6-BA groups was calculated using the Wilcoxon *t*-test (without *, not significant; * *p* < 0.05; ** *p* < 0.01; *** *p* < 0.001). (**B**) Schematic diagram of gene regulation patterns in floral development of *Bougainvillea*. Red circle indicates the differentiated floral bud tissue located at the apical of thorn. The question mark indicates that the regulatory pathway is unclear. *GI*, *GIGANTEA*; *FT*, *FLOWERING LOCUS T*; *CO*, *CONSTANS*; *FLC*, *FLOWERING LOCUS C*; *FD*, *FLOWERING LOCUS D*; *LFY*, *LEAFY*; *SPL*, *SQUAMOSA PROMOTER-BINDING-LIKE*; *SOC1*, *SUPPRESSOR OF OVEREXPRESSION OF CONSTANS 1*.

We identified that many homologous genes related to the flowering pathways were upregulated under 6-BA application (Supplementary Tables S7 and S8) and mainly clustered in light-dependent, age, and gibberellin pathways. In the light-dependent pathway, *CONSTANS* (*CO*) is the key downstream gene that positively regulates flowering in *Arabidopsis* and rice, which are the typical representatives of SD and long-day (LD) flowering plants, respectively [22,23,24]. In *Arabidopsis*, the *CO*-*FT* module in vascular bundles regulates flowering under normal LD conditions, whereas *GI* of mesophyll or vascular tissues directly activates *FT* to promote flowering without upregulating *CO* under SD conditions [25]. We observed that *GI* and *FT* expressions were upregulated in the leaves of *B. glabra* ‘Sao Paulo’ under 6-BA treatment (Figure 5), suggesting their potential synergistic effect in promoting early flowering under non-inductive LD conditions. Moreover, age and gibberellin pathways also play important roles in flowering in parallel with photoperiodic pathways. Previous studies have indicated that *SPL9* and *SPL3* induce flowering via activating MADS-box genes of *AP1*, *LFY*, *FUL*, and *SOC1*, while *SPL13* can directly activate *FT* to regulate inflorescence development positively [26,27]. In early-flowering *Bougainvillea*, many *SPL* genes comprising *SPL4*, *SPL9*, and *SPL13* were upregulated, which is consistent with previous genetic and biochemical evidence in *Arabidopsis*, loquat, and tomato [27,28,29], suggesting that *SPLs* probably promote the phase transition from vegetative to floral meristems in *Bougainvillea*. Previous studies showed that application of gibberellin (GA) biosynthesis inhibitors, such as chlormequat, daminozide, and paclobutrazol, promoted flowering in *Bougainvillea* [30,31,32]. Consistent with these results, our other experiments demonstrated that exogenous GA application enhanced shoot elongation but suppressed flowering in *Bougainvillea* (Supplementary Figure S8). Correspondingly, the expression of *gibberellin 20-oxidase (GA20ox*), the key GA biosynthesis gene, showed no significant difference under 6-BA treatment in this study. Meanwhile, we observed that one gene of GA-activating enzyme (*gibberellin 3-beta-dioxygenase*, *GA3ox*) was significantly downregulated, and two genes of GA-deactivating enzyme (*gibberellin 2-beta-dioxygenase*, *GA2ox*) were significantly upregulated (Supplementary Table S3). However, there are other *GA3ox* and *GA2ox* genes that exhibited opposite expression patterns, respectively. Therefore, we could not conclusively determine whether 6-BA treatment affected GA activity. Furthermore, we also found that DELLA, the GA perception protein, showed downregulation in the 6-BA treatment process, which is consistent with its function of repression of flowering. DELLA protein can directly bind to CO to downregulate *FT* [33,34] or inhibit *SOC1* and *LFY* to delay flowering [35]. Therefore, we speculate that 6-BA treatment may regulate *Bougainvillea* flowering through affecting GA signaling transduction rather than GA biosynthesis. In flowering plants, *FT* has been recognized as florigen, which is synthesized in leaves and transported to the apical meristem for flower inducing [36,37,38,39]. In this experiment, we also found that expression of *FT* significantly increased in the leaves of the 6-BA treatment (Figure 5A). Previous studies have shown that those upstream regulatory pathways transmit signals to the downstream flower meristem via integration genes *FT* and *SOC1*, thereby determining flower bud differentiation [21].

The *WUSCHEL-related homeobox* (*WOX*) genes within the homeodomain transcription factor family have been demonstrated to serve as key regulators in maintaining stem cell homeostasis [40] and function as direct downstream effectors of cytokinin signaling pathways [41]. During apple floral transition after 6-BA treatment, *WOX3* exhibited a high expression level and potentially mediated cytokinin signaling to promote floral bud differentiation [42]. Consistent with these findings, we observed significant upregulation of the *WOX3* gene during 6-BA-induced flowering in *Bougainvillea*, suggesting that *WOX3* may perform an evolutionarily conserved function in transducing cytokinin signals to stimulate floral bud formation and development.

It is notable that the role of *FLC* in regulating the flowering process of *Bougainvillea* may differ from our common understanding. In *Arabidopsis*, *FLC* inhibits the expression of downstream flowering integrator genes *FT* and *SOC1*, thereby suppressing flowering [43]. In certain plant species, transitions from vegetative to reproductive phases require prolonged exposure to cold temperatures, which is known as vernalization. The floral repressor FLC is the key regulator in vernalization and is stably inhibited and modified by *VRN* genes [44]. In *Arabidopsis*, *VRN1* acts not only as a flowering time regulator in response to vernalization, but also performs a vernalization-independent function by accelerating flowering through positively regulating floral integrator *FT* [45]. Interestingly, we found that the B3 domain-containing transcription factor *VRN1* (*Bou_88844*), homologous to *Arabidopsis VRN1* gene, was upregulated during floral development in *Bougainvillea*. Considering the absence of vernalization in *Bougainvillea*, *VRN1* may regulate flowering by other pathways in this species. In contrast to its conserved flowering-repressing function in other species, *FLC* expression was significantly upregulated in *Bougainvillea* under 6-BA treatment, along with early flowering of *Bougainvillea*. It suggests that *FLC* may play a different role in the flowering regulation of *Bougainvillea*, but the underlying mechanism requires further exploration.

In plants, histone modifications comprising methylation, acetylation, phosphorylation, ubiquitylation, ADP-ribosylation, sumoylation, carbonylation, and biotinylation, serve as key epigenetic mechanisms that modulate chromatin structure and gene transcriptional activity [46]. Currently, multiple histone methyltransferases have been found to interact with genes such as *FLC*, *FT*, *CO*, and *SOC1*, thereby regulating the flowering process [47]. We observed significant upregulation of several histone methyltransferases in the 6-BA treatment group (Supplementary Table S3), though their potential roles in flowering regulation remain unreported. Additionally, transcriptome analysis revealed that *histone deacetylase 15* (*HDA15)* was significantly upregulated during the flowering process of *Bougainvillea* (Supplementary Table S3). Histone deacetylation enhances histone–DNA binding by tightening nucleosome structure, thereby suppressing gene transcription. In *Arabidopsis*, *HDA15* delayed leaf senescence and flowering, concomitant with *FLC* upregulation and *FT* downregulation [48]. Similarly, *HDA15* may participate in flowering regulation by modulating the expression of *FLC* and *FT* genes in *Bougainvillea.*

Based on these results and previously published studies, we confirm that 6-BA can promote the flowering of *Bougainvillea* and speculate a conceptual model for the regulatory network of the hormonal effects on flower development in *Bougainvillea* (Figure 5B). This study proposes that 6-BA promotes floral bud differentiation and development in *Bougainvillea* through a hierarchical regulatory network. According to the model, exogenous 6-BA enters specific tissues (particularly thorns) and activates the endogenous cytokinin signaling pathway. Subsequently, this signal coregulates a network of the photoperiod pathway (e.g., *COL*), flowering integrators (e.g., *FT*, *SOC1*), and floral meristem identity genes (e.g., *LFY*), in a process coordinated by the SPL-mediated pathway to collectively drive floral bud development.

To validate this model, future research should prioritize the functional characterization of core transcription factors (e.g., *FLC*, *LFY*, *SPL*) in *Bougainvillea*, through transgenic approaches (overexpression or knockdown) to directly assess their impact on floral initiation. Given the lack of a stable transformation system in Bougainvillea, heterologous expression in model plants such as Arabidopsis or Petunia is recommended. Furthermore, the protein–protein interactions between 6-BA signaling components and these transcription factors should be identified using yeast two-hybrid or co-immunoprecipitation techniques to elucidate the specific molecular connections within the regulatory network.

## Data Availability

All raw reads of RNA sequencing were deposited in the National Center for Biotechnology and Information (NCBI) short-read archive repository under the accession numbers SAMN44525952-SAMN44525957 (BioProject accession: PRJNA1180694).

## Supplementary Materials

The following supporting information can be downloaded at: https://www.mdpi.com/article/10.3390/plants14223442/s1. Table S1. PCR primer sequences used in this study. Table S2. Statistics of RNA sequencing data from thorns of 6-BA treatment and control group. Table S3. DEGs obtained by comparasion with 6-BA and CK group. Table S4. GO terms enriched for up-regulated DEGs (differentially expressed genes) between 6-BA and CK group. Table S5. KEGG terms enriched for differentially expressed genes between 6-BA and CK group. Table S6. GO terms enriched for down-regulated DEGs between 6-BA and CK group. Table S7. Up-regulated genes and transcription factors (TFs) enriched in flower development under 6-BA treatment. Table S8. Up-regulated genes enriched in photoperiodism regulation of flowering under 6-BA treatment. Figure S1: Inflorescence morphology of *B. glabra* ‘Sao Paulo’. (A) Floral unit of a single flower subtended by a bract. (B) Primary cymose inflorescence. (C) Secondary cymose inflorescence. (D) Cymose panicles. Figure S2: The correlation analysis of RNA sequencing samples between CK and 6-BA groups. Figure S3: Heatmap cluster of upregulated DEGs enriched in photoperiodism regulation of flowering under 6-BA treatment. Figure S4: Validation of the expression of flowering-related genes using qPCR analysis. Blue and purple bar charts indicate gene relative expression of CK and 6-BA group, respectively, on the left side of each diagram. Blue and purple bar charts indicate gene expression values of log_2_(FPKM +1) of CK and 6-BA group, respectively, on the right side of each diagram. Error bars represent the standard deviation of three biological replicates. Statistical significance between CK and 6-BA group was calculated using the Wilcoxon *t*-test (without *, not significant; * *p* < 0.05; ** *p* < 0.01; *** *p* < 0.001; **** *p* < 0.0001). Figure S5: Neighbor-joining tree analysis of Bou_64595 and other homologous FLOWERING LOCUS C proteins downloaded from NCBI database. Figure S6: Neighbor-joining tree analysis of Bou_913 and other homologous FLOWERING LOCUS T proteins downloaded from NCBI database. Figure S7: Neighbor-joining tree analysis of Bou_19129 and SPL proteins downloaded from *Arabidopsis thaliana* TAIR database (http://www.arabidopsis.org). Figure S8: Phenotypic effects of GA treatment on *B. glabra* ‘Sao Paulo’. (A) Overall branch morphology of CK- (left) and GA-treated (right) plants. (B) The thorns of CK (left) and GA (right) groups. The red circles highlight the eighth thorn from the apical bud, with the area within the white box shown in the enlarged inset. Both CK- and GA-treated samples display wilting at the thorn apex. (C) The leaves of CK (left) and GA (right) groups.
