# Supplementary material for: Comparative Transcriptome Analysis Provides Insight into the Effect of 6-BA on Flower Development and Flowering in Bougainvillea
Source: Plants (Basel). 2025 Nov 10;14(22):3442. doi: 10.3390/plants14223442 (PMC12656518; doi:10.3390/plants14223442)
Supplement: Supplementary file 1 [file plants-14-03442-s001.zip › Supplementary Figures/Supplementary Figure S3 Heatmap cluster of up-regulated DEGs enriched in photoperiodism regulation of flowering under 6-BA treatment.pdf]

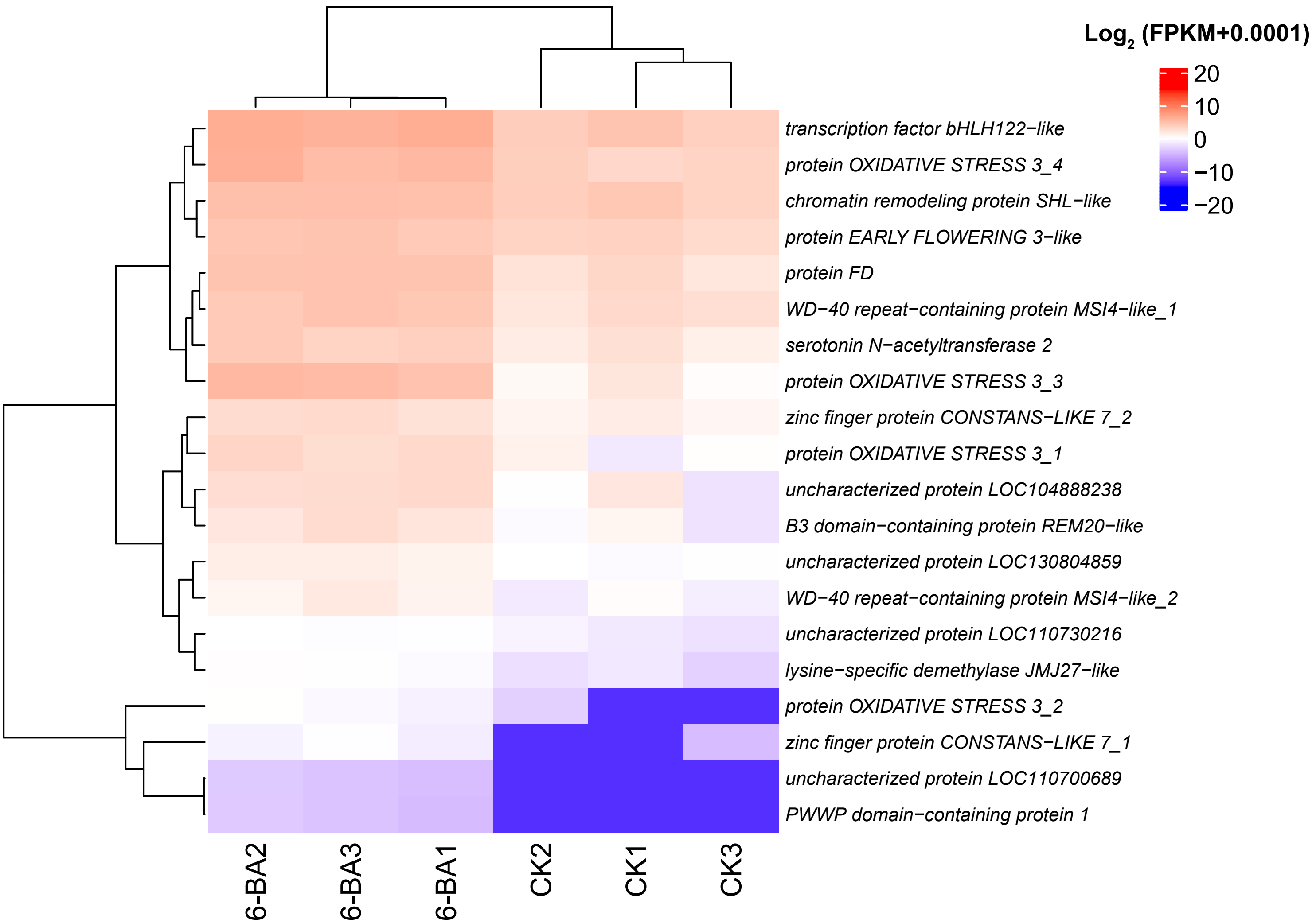

Supplementary Figure S3 Heatmap cluster of up-regulated DEGs enriched in photoperiodism regulation of flowering under 6-BA treatment.
