# Supplementary material for: Comparative Transcriptome Analysis Provides Insight into the Effect of 6-BA on Flower Development and Flowering in Bougainvillea
Source: Plants (Basel). 2025 Nov 10;14(22):3442. doi: 10.3390/plants14223442 (PMC12656518; doi:10.3390/plants14223442)
Supplement: Supplementary file 1 [file plants-14-03442-s001.zip › Supplementary Figures/Supplementary Figure S4 Validation of the expression of flowering-related genes using qRT-PCR analysis3.pdf]

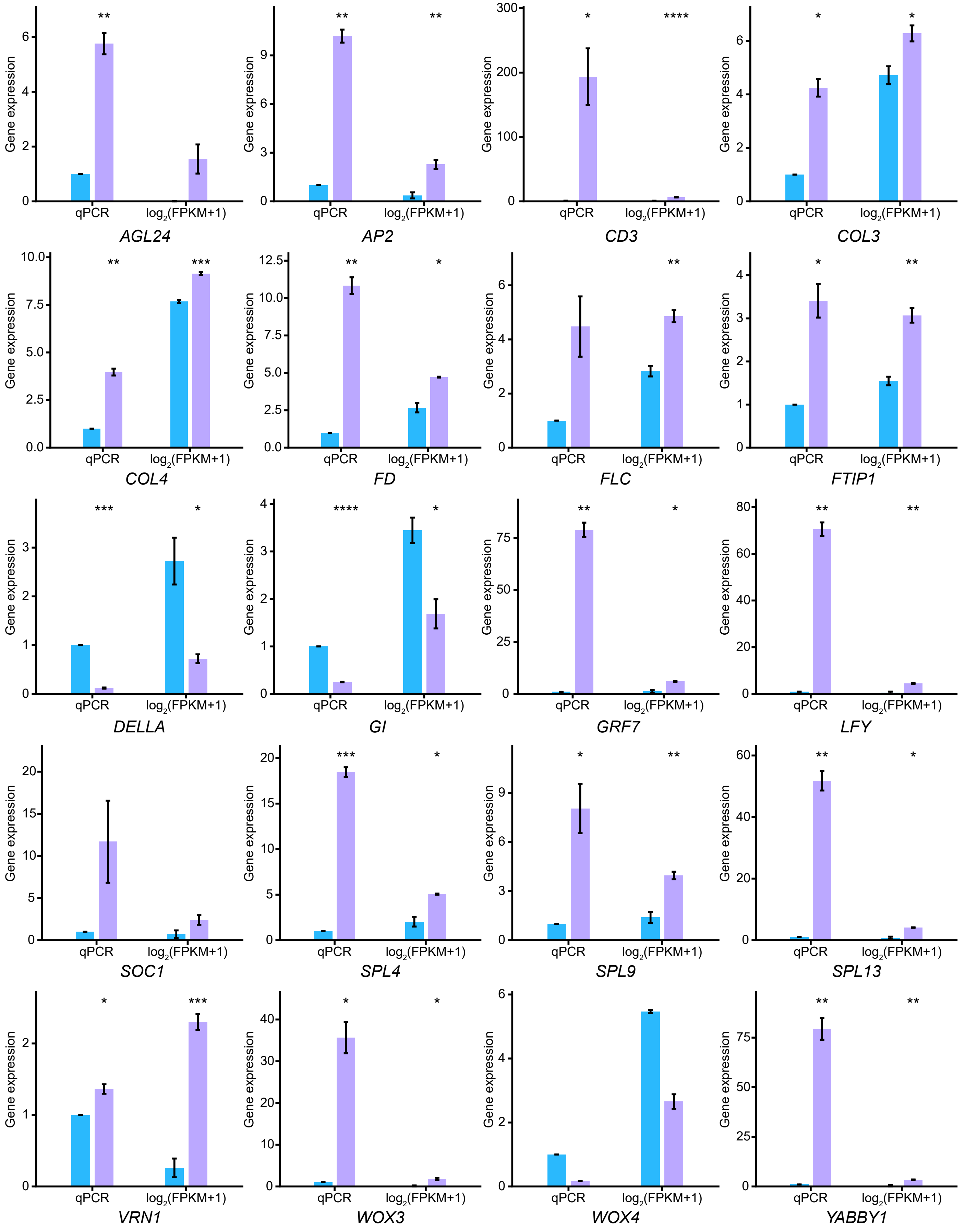

Supplementary Figure S4 Validation of the expression of flowering-related genes using qRT-PCR analysis. Blue and purple bar charts indicate gene relative expression of CK and 6-BA group respectively on the left side of each diagram. Blue and purple bar charts indicate gene expression values of log_2(FPKM +1) of CK and 6-BA group respectively on the right side of each diagram. Error bars represent the standard deviation of three biological replicates. Statistical significance between CK and 6-BA group was calculated using the wilcoxon t.test (without *, not significant; * $P < 0.05$; ** $P < 0.01$; *** $P < 0.001$; ****$P < 0.0001$).
