# Supplementary material for: Comparative Transcriptome Analysis Provides Insight into the Effect of 6-BA on Flower Development and Flowering in Bougainvillea
Source: Plants (Basel). 2025 Nov 10;14(22):3442. doi: 10.3390/plants14223442 (PMC12656518; doi:10.3390/plants14223442)
Supplement: Supplementary file 1 [file plants-14-03442-s001.zip › Supplementary Figures/Supplementary Figure S5 Neighbor-Joining tree analysis of Bou_64595 and other homologous FLOWERING LOCUS C proteins.pdf]

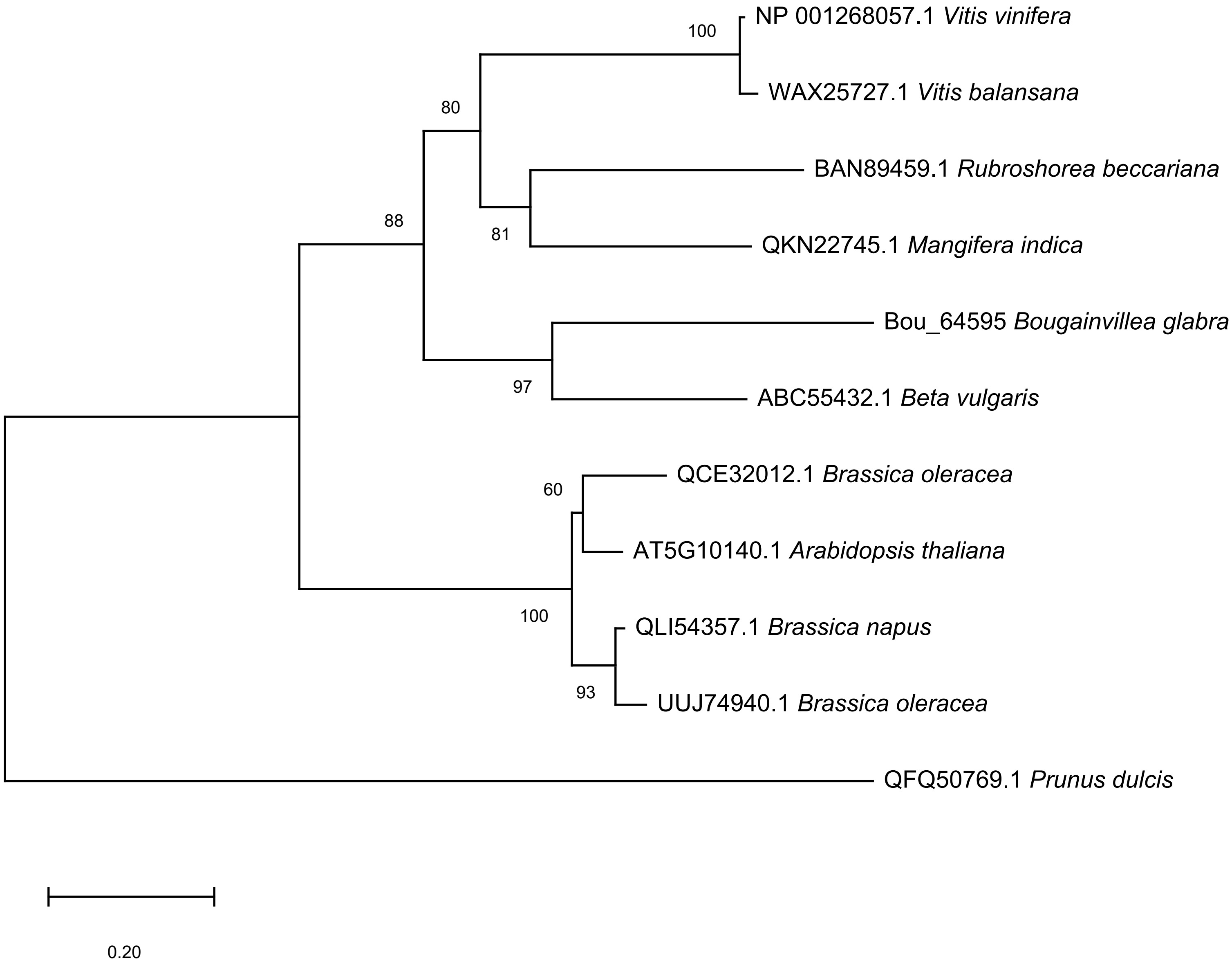

Supplementary Figure S5 Neighbor-Joining tree analysis of Bou_64595 and other homologous FLOWERING LOCUS C proteins downloaded from NCBI database.
