# Supplementary material for: Comparative Transcriptome Analysis Provides Insight into the Effect of 6-BA on Flower Development and Flowering in Bougainvillea
Source: Plants (Basel). 2025 Nov 10;14(22):3442. doi: 10.3390/plants14223442 (PMC12656518; doi:10.3390/plants14223442)
Supplement: Supplementary file 1 [file plants-14-03442-s001.zip › Supplementary Figures/Supplementary Figure S7 Neighbor-Joining tree analysis of Bou_19129 and SPL proteins.pdf]

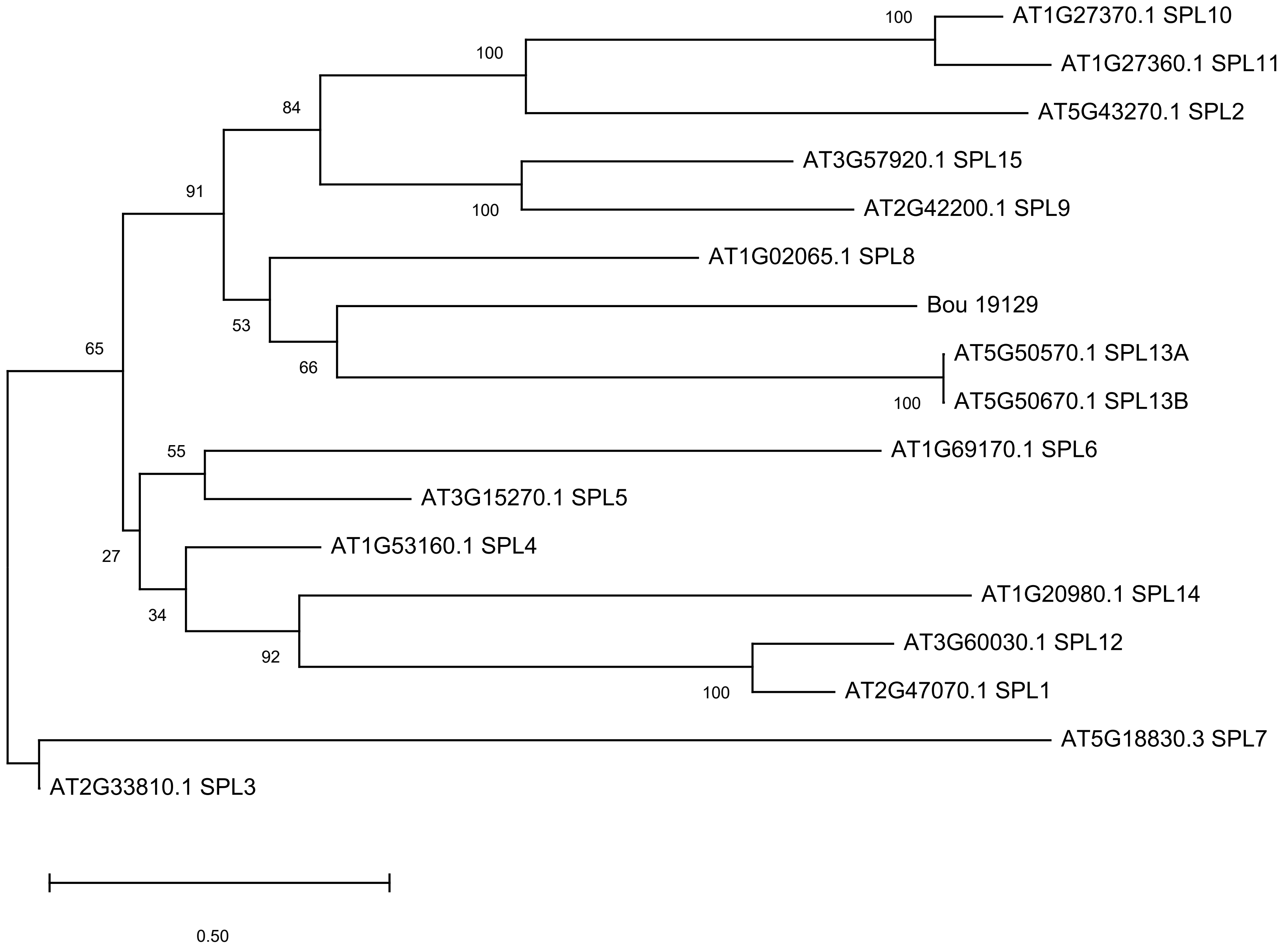

Supplementary Figure S7 Neighbor-Joining tree analysis of Bou_19129 and SPL proteins downloaded from *Arabidopsis thaliana* TAIR database (http://www.arabidopsis.org).
