# Supplementary material for: Comparative Transcriptome Analysis Provides Insight into the Effect of 6-BA on Flower Development and Flowering in Bougainvillea
Source: Plants (Basel). 2025 Nov 10;14(22):3442. doi: 10.3390/plants14223442 (PMC12656518; doi:10.3390/plants14223442)
Supplement: Supplementary file 1 [file plants-14-03442-s001.zip › Supplementary Figures/Supplementary Figure S8 Phenotypic effects of GA treatment on B. glabra 'Sao Paulo'.pdf]

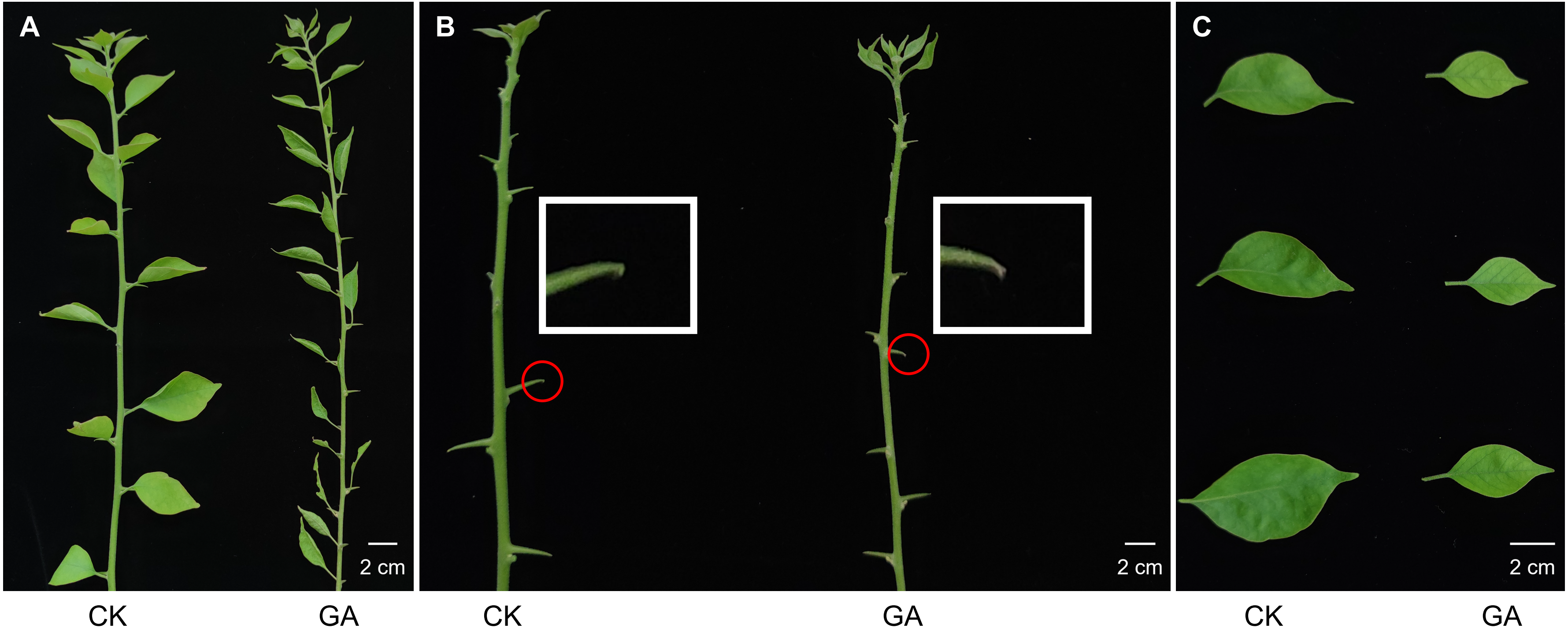

Supplementary Figure S8 Phenotypic effects of GA treatment on *B. glabra* 'Sao Paulo'. **(A)** Overall branch morphology of CK (left) and GA-treated (right) plants. **(B)** The thorns of CK (left) and GA (right) groups. The red circles highlight the eighth thorn from the apical bud, with the area within the white box shown in the enlarged inset. Both CK and GA-treated samples display wilting at the thorn apex. **(C)** The leaves of CK (left) and GA (right) groups.
